# Editorial: Advances and Obstacles in Contemporary Nonverbal Communication Research

**DOI:** 10.3389/fpsyg.2021.731334

**Published:** 2021-08-12

**Authors:** Miles L. Patterson, Norah E. Dunbar, Marianne Schmid Mast, J. M. Fernandez-Dols

**Affiliations:** ^1^Department of Psychological Sciences, University of Missouri-St. Louis, St. Louis, MO, United States; ^2^Department of Communication, University of California, Santa Barbara, Santa Barbara, CA, United States; ^3^Department of Organizational Behavior, Faculty of Business and Economics, University of Lausanne, Lausanne, Switzerland; ^4^Facultad de Psicología, Universidad Autónoma de Madrid, Madrid, Spain

**Keywords:** nonverbal communication, facial expression, culture, technology, deception

For centuries, speculation about the meaning and impact of nonverbal behavior has been common in literature, philosophy, and science (see Knapp, 2006 for a historical review). In the latter half of the nineteenth century, Darwin's 1872 *The expression of the emotions in man and animals* work was particularly instrumental in focusing attention on expressive behavior. Nevertheless, sustained and systematic empirical research on nonverbal communication was not widespread until the middle of the twentieth century. Examples of its diverse roots can be found in anthropology (Birdwhistell, 1955, 1970; Hall, 1959, 1966), sociology (Goffman, 1959, 1963), and psychology (Sommer, 1959, 1962; Exline, 1963; Ekman, 1964, 1965). Since that time, literally tens of thousands of articles and hundreds of scholarly books have expanded our knowledge of the nonverbal communication and prompted new and interesting questions about its scope and functions. This acceleration of publications, especially in recent years, provides an appropriate opportunity to examine the current landscape of nonverbal communication research and to provide an outlook into future areas and topics.

In laying the foundation for our “Advances and Obstacles” issue, it is worth noting some of the important topics addressed in current research. For example, we are learning more about the accuracy of pervasive automatic judgments of others' appearance and behavior (Todorov, 2017; Murphy et al., 2019). But automatic judgments can also facilitate prejudice and discrimination, as studies of implicit bias show (Richeson and Shelton, 2005). The long-held view that facial expressions necessarily reflect underlying emotions (Ekman, 1982) is now being challenged. One alternative view proposes that facial behaviors are adaptive and adaptable tools for social influence, rather than universal uniform expressions of basic emotions (Crivelli and Fridlund, 2018). The relative merits of these opposing views also have relevance for understanding nonverbal communication in a variety of settings, including the justice system (e.g., detecting deception), policy decisions, national security, and clinical settings (Denault et al., 2020). Research on cultural differences in nonverbal communication provides insight into cultural dynamics and is relevant for reducing inter-group conflict and facilitating cooperation (Matsumoto and Hwang, 2016). Exciting recent work in behavioral neuroscience examines the neural correlates of nonverbal communication (e.g., Jacob et al., 2014, Lindenberg et al., 2012; Arioli and Canessa, 2019).

In the present digital age, rapidly-evolving communication technologies might seem to displace the more mundane role of face-to-face nonverbal communication in everyday life. The continuing expansion of social media, artificial intelligence systems, virtual reality, and social robots, however, is not replacing, but rather extending nonverbal communication to new platforms (see also von der Pütten et al., 2010; Hasler and Friedman, 2012; Küster et al., 2015; Patterson, 2019; Blunden and Brodsky, 2021). As a result, this is a time of expanding research and theory into new domains. Nevertheless, the opportunities provided by the new technologies must be weighed against the ease of spreading misleading and deceptive images that affect our trust in their content (e.g., Tolosana et al., 2020).

Consequently, this is an appropriate time to (1) examine more fully the questions driving current research and theory, (2) weigh the obstacles to a broader understanding of nonverbal communication, and (3) consider the potential opportunities for advancing future research on nonverbal communication. The collection of articles here is testimony to the diversity of nonverbal communication research in addressing these goals.

Many of the 17 articles in this issue focus in some fashion on methodological advances and their potential limitations in new directions for research. Murphy and Hall review the thin-slice method with a particular focus on its reliability and validity in representing sustained behavioral sequences. The article proposes that deciding if and when to employ thin-slice measurement should focus on its broader representativeness for behavior, predictive validity for variables or constructs beyond the sampled behavior, and assessing how the length of the sampled thin-slices affects the accuracy of interpersonal judgments.

Three articles deal with new technologies that include machine learning and the application of algorithms to the scoring and evaluation of nonverbal stimuli. Albohn and Adams applied computer vision algorithms to the structure, color, and texture of faces to predict gender-stereotypic impressions. In addition, the computer impressions were similar to those made by human participants. The broader issue of the opportunities and limitations of machine learning were addressed in two other articles. Burgoon et al. used machine learning and automated analysis to examine the role of dominance-submission, composure-nervousness, and trust-mistrust in relational communication. They also discussed the potential benefits of the new techniques in simplifying the study of nonverbal communication. Renier et al. also recognize the utility of applying algorithms in machine learning techniques in analyzing nonverbal behavior. Nevertheless, they caution that automated nonverbal coding can be as biased as human coding and can be limited to the particular context for the behavior.

Several empirical articles focus on a variety of issues related to the encoding and decoding of expressive displays. Bente et al. developed a motion capture and character animation method eliminating cultural and gender appearance cues that can precipitate stereotypic biased judgments. In the absence of visual culture and gender cues, they found that female dyads were rated significantly higher on rapport and that this difference was greater in Arab dyads than in German dyads. Song et al. examined anger and sadness expressions in South Korean and American samples. They found that in both cultures, anger and sadness displays signaled both negative and positive underlying states. Fugate and Franco studied the correspondence between human facial expressions and analogous emoji faces. They found that the majority of emoji faces did not conform to human emotional expressions, even though the anatomical codes for the two types of faces were generally shared. Etcoff et al. investigated the effects of botulinum toxin treatments on the perceptions of pre- and post-treatment smiles. Pre-treatment smiles were rated as more felt, more spontaneous, and happier than post-treatment smiles. Although post-treatment patients were rated as looking younger, they were not judged as more attractive than pre-treatment patients. The effects of tears on visual attention to faces and on subsequent judgments of emotional intensity were the focus of an experimental study by Pico et al. An eye tracking method provided evidence for tears being a magnet for visual attention that, in turn, facilitated perceptions of greater emotional intensity. Ruben et al. addressed the issue of whether technology use enhanced or hindered nonverbal decoding skill. Overall screen time was unrelated to objective measures of decoding skill, but how participants used their screen time was related to decoding skill. Active users (e.g., posting content) performed worse on decoding skill measures, but passive users performed better.

Various issues dealing with authenticity/deception in expressive behavior are the focus of three other articles. Zloteanu and Krumhuber discuss different perspectives on facial displays in the context of increasing evidence contradicting the traditional view that reliable facial muscle movements signal distinct emotional experiences. They discuss spontaneous vs. posed expressions and advocate a functional approach to expressions as neurophysiological states and communicative signals. Vrij and Fisher's article addresses the common assumption that liars display more nervous behaviors than truth tellers. They provide evidence that liars do not show more nervous behaviors. Consequently, observers who focus on such nervous behaviors are likely to do poorly in detecting deception. On a similar theme, Denault discusses the negative consequences of depending on unreliable nonverbal cues for detecting deception. Specifically, in the justice system, judges, and juries are vulnerable to the common, but scientifically discredited, assumption of valid nonverbal indicators of deception. As a result, assessments of witness credibility can be distorted, with detrimental effects on trial outcomes.

The last four articles provide a range of commentaries on approaches to future research. Matsumoto and Hwang advocated for a multimodal approach to research and theory. That is, increased attention to clusters of nonverbal behavior, rather than a single channel at a time, can facilitate our understanding of underlying mental states. Carrard addresses a similar theme of linking interactants' inner preferences and expectations to patterns of nonverbal behavior. That is, nonverbal communication should be viewed as an adaptive process driven by actors' inner characteristics. DeGroot et al. focus on the emerging and important research on the diverse effects of olfaction on a wide variety of interpersonal processes, including identity, emotion, and mate selection. The authors argue that pursuing effectively the wide range of important issues in olfaction requires an integration of the psychology and chemistry disciplines into a new field of “sociochemistry.” Finally, Kirkwood et al. extend the process of interpersonal synchrony from the nonverbal mimicry between partners to individuals' synchrony with wearable exoskeletons. Recent technological advances in wearable robots are designed to augment a user's strength and mobility. The authors discuss the utility of the Interpersonal Adaptation Theory in facilitating research maximizing human-exoskeleton synchrony.

In conclusion, we hope that this interesting set of articles provides an informative window into some of the diverse issues driving current research on nonverbal communication. The advances in research discussed in many of these articles are often responses to existing obstacles or discrepancies in research. Other articles are focused more on identifying the new obstacles yet to receive attention that, in turn, will stimulate new research. Thus, the present issue provides a vehicle for facilitating our understanding of nonverbal communication and appreciating where future research may be headed.

## Author Contributions

All of the authors have contributed to this work and approved its publication.

## Conflict of Interest

The authors declare that the research was conducted in the absence of any commercial or financial relationships that could be construed as a potential conflict of interest.

## Publisher's Note

All claims expressed in this article are solely those of the authors and do not necessarily represent those of their affiliated organizations, or those of the publisher, the editors and the reviewers. Any product that may be evaluated in this article, or claim that may be made by its manufacturer, is not guaranteed or endorsed by the publisher.
